# Stereolithographic 3D printing of extrinsically self-healing composites

**DOI:** 10.1038/s41598-018-36828-9

**Published:** 2019-01-23

**Authors:** Polly Sanders, Adam J. Young, Yang Qin, Kevin S. Fancey, Michael R. Reithofer, Rémy Guillet-Nicolas, Freddy Kleitz, Nicole Pamme, Jia Min Chin

**Affiliations:** 1 Department of Chemistry, University of Hull, Cottingham Road, Hull, HU6 7RX United Kingdom; 2 Department of Inorganic Chemistry, Faculty of Chemistry, University of Vienna, Währinger Straße 42, 1090 Vienna, Austria; 3 Department of Inorganic Chemistry – Functional Materials, Faculty of Chemistry, University of Vienna, Währinger Straße 42, 1090 Vienna, Austria

## Abstract

We demonstrate for the first time the direct stereolithographic 3D printing of an extrinsically self-healing composite, comprised of commercial photocurable resin modified with anisole and PMMA-filled microcapsules. The composites demonstrate solvent-welding based autonomous self-healing to afford 87% recovery of the initial critical toughness. This work illustrates the potential of stereolithographic printing to fabricate self-healing composites with user-defined structures, avoiding the need for extensive rheological optimization of printing inks, like in direct-write 3D printing. Importantly, this work also demonstrates the inclusion of microcapsules into 3D printing resins to incorporate additional functionality into printed composites, which could be adapted for applications beyond self-healing materials.

## Introduction

The lifetimes of composite materials are typically limited by fatigue or other material failure mechanisms due to damage encountered during service. However, in nature, plants and animals overcome this limitation by utilizing self-healing as a crucial survival strategy to repair damage to their tissues. Taking lessons from nature, scientists have extensively researched the incorporation of self-healing capabilities into synthetic polymeric materials to prolong their operational lifetimes^1–5^.

Self-healing materials are classified into two categories – intrinsic and extrinsic. Intrinsic self-healing materials rely on reversible bonds such as metal-ligand bonds^6^ or hydrogen bonds^7^ to facilitate healing and are therefore typically limited to gels or elastomeric materials which allow for molecular diffusion^1^. Extrinsic self-healing, however, utilizes healing components sequestered from the main matrix within microcapsules or vascular networks^2^. During composite fracture, the capsules or network are broken, releasing the healing agents which react with each other or interact with the matrix to seal the fracture. In this case, stiff polymer matrices can be used, as molecular diffusion of the matrix is not a requirement for healing. Therefore, extrinsic self-healing is desirable for many practical applications requiring hard polymeric structures.

In parallel, the use of 3D printing (3DP) has become increasingly ubiquitous in different fields due to the ability to generate user-defined 3D objects with a variety of materials^8^. 3DP encompasses a family of additive manufacturing techniques that allow rapid yet flexible fabrication of complex 3D structures with features from the sub-micron to the multi-meter scale. Materials such as ceramics^9^, resins^10^ and even novel nanocomposites^11^ can be precisely structured through 3DP. Direct 3DP of elastomers and hydrogels exhibiting intrinsically self-healing properties has also been demonstrated^12–14^. Researchers have also 3D printed sacrificial scaffolds, which are then utilized for templating vascular self-healing systems. The printed scaffolds are embedded into a polymeric matrix which is cured, then the scaffold is removed via washing or heating under vacuum, and replaced with healing agents. The vascular ends are then sealed to afford the resulting self-healing composite^15,16^. However, to our knowledge, the direct 3D printing of an extrinsically self-healing system has not been reported.

## Results and Discussion

**Figure 1:**
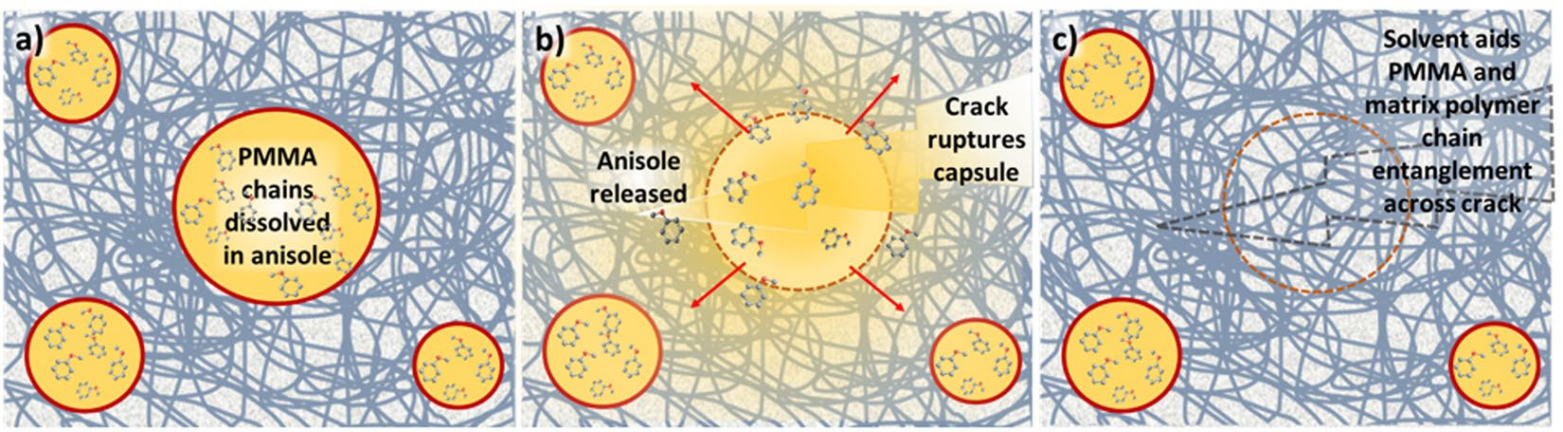
Schematic illustration of the solvent welding based self-healing mechanism: (**a**) The virgin material with intact microcapsules embedded within the polymer matrix; (**b**) crack propagation and rupture of the microcapsule shell. The encapsulated solvent anisole diffuses into the surrounding polymer matrix. This enhances polymer diffusion across the crack and polymer chain entanglement; (**c**) polymer chain entanglement heals the crack.

Here, we demonstrate a technique of combining UV-curable resin embedded with solvent-containing microcapsules in conjunction with stereolithographic (SL) 3DP to construct user-defined 3D structures, whereby a laser (405 nm) spatioselectively polymerizes/crosslinks the resins according to a computer aided design. The self-healing employed in this work follows a solvent welding mechanism, as illustrated in Fig. 1. When a crack occurs and ruptures a capsule along the propagation pathway, the solvent within the capsule is released into the matrix. Solvent release promotes polymer diffusion and entanglement across cracks formed in the matrix, leading to crack healing^17,18^. Such a method is advantageous in its simplicity and cost-effectiveness, with no need for expensive metal catalysts^19^ or the preparation of multiple types of microcapsules containing different healing reagents^20^.

Anisole, which is widely used in both the fragrance industry and as a food additive, was selected as the solvent for encapsulation due to its low toxicity^17^. The high boiling point and immiscibility of anisole with water also allows it to be easily encapsulated using *in situ* polymerization of urea-formaldehyde in an oil-in-water emulsion^21^. Importantly, anisole has been shown to be a suitable solvent for solvent welding based self-healing in PMMA^22,23^ which contains methacrylate functionalities commonly found in most commercially available SL 3DP resins. Anisole was therefore expected to also be a good candidate for healing photocured SL 3DP resins and preliminary testing showed that anisole could soften and increase tackiness of surfaces in such photocured samples. Anisole-containing capsules were prepared using a technique modified from that described by Brown^21^. This afforded urea-formaldehyde microcapsules with an anisole and 5 wt% PMMA core, which had an average diameter of 130 ± 15 µm (Figs S1 and S2). PMMA was incorporated into the microcapsules together with anisole, as the work by Gladman *et al*. showed that the inclusion of PMMA improved healing efficiencies^23^.

**Figure 2:**
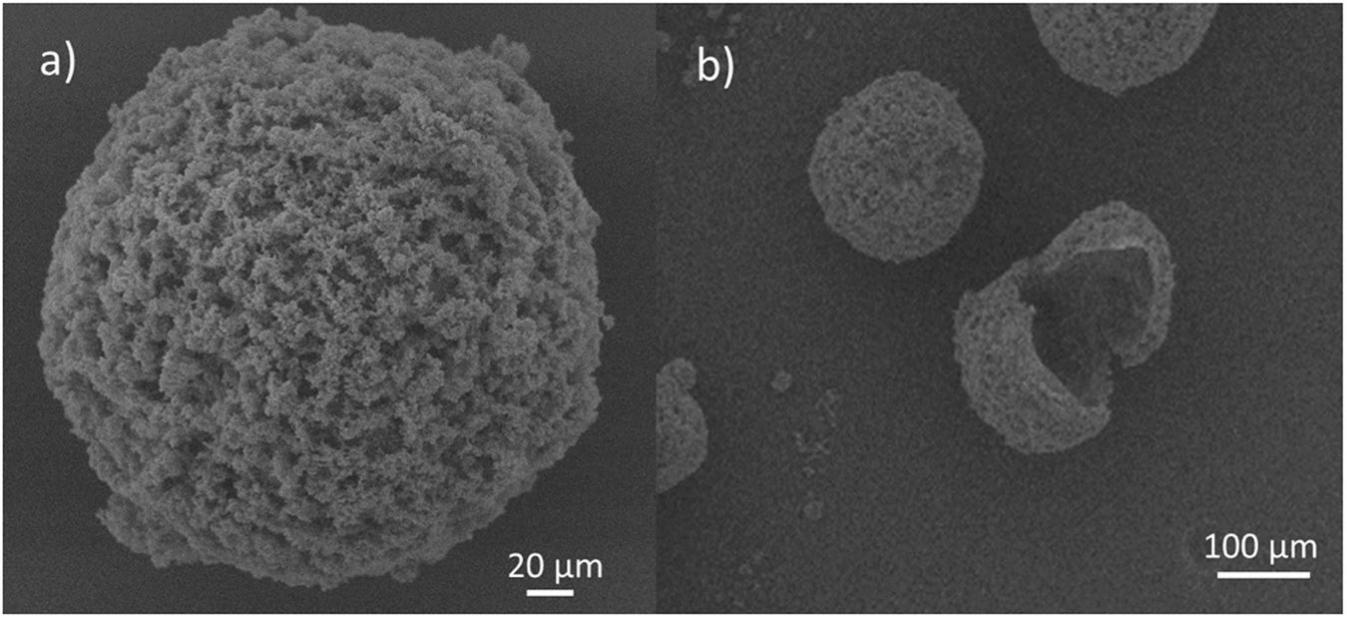
Representative SEM images showing (**a**) the rough surface of a urea-formaldehyde microcapsule shell; (**b**) a ruptured capsule showing the cross-section of its shell wall.

Scanning electron microscopy (SEM) (Fig. 2) showed that the microcapsule walls possessed a rough surface, similar to observations reported by others^21,24^. The capsule roughness has been attributed to the precipitation of polymerized urea-formaldehyde from the water phase and its deposition onto the capsule wall at the oil-water interface during *in situ* polymerization. This shell roughness is desirable as it promotes capsule adhesion to the polymer matrix and provides a greater possibility for microcapsule rupture in the event of crack propagation^21^.

The microcapsules were mixed into the UV-curable resin to achieve 2.5, 5 and 10 wt% capsule concentrations. SEM showed that the capsules were successfully embedded into the cured polymer matrix (Fig. S3). FTIR spectroscopy was also performed on the cured composites (Figs S4, S5 and S6), whereby only the composites with capsules showed the presence of the urea stretching mode at 3410 cm^−1^. Thermogravimetric analysis (TGA) of anisole/PMMA-filled urea-formaldehyde microcapsules showed a sudden rupture of the capsules at approximately 260 °C. Repeating this experiment twice more, we again observed this phenomenon each time. This was attributed to the explosion of the capsules upon sufficient thermal degradation of the urea-formaldehyde shell, which occurs between 220–300 °C^25,26^ and internal capsule pressure arising from vaporization of anisole and PMMA degradation products. However, TGA of microcapsules in cured resin mixtures did not show a similar rupture event, presumably as the presence of the resin matrix prevented explosion of microcapsules (Fig. S7).

To investigate their self-healing properties, mode 1 fracture testing was performed on tapered double cantilever beam (TDCB) test samples comprising of these mixtures (Fig. S8), which were generated using a molding technique. Degassed microcapsule-resin mixtures were poured into silicone molds and UV cured. The self-healing efficiency of anisole/PMMA microcapsule composites was quantified using a protocol for extrinsically self-healing materials first utilized by White *et al*^27^. This approach defines the self-healing efficiency of the material as a ratio of the fracture toughness, *K*_*c*_, of the virgin material versus that of the healed material. *K*_*c*_ is linked to the critical load, *P*_*c*_ (the load at which crack propagation occurs) as shown in equation (1).

1 $${K}_{c}=\alpha {P}_{c}$$

Here, *α* is a geometric constant specific to the host matrix. However, this is complicated by the heavy reliance of *P*_*c*_ on initial crack length, as the initial crack lengths of the virgin samples may differ from that of the healed samples. The use of samples with TDCB geometry allows us to overcome this complication. In samples with this geometry, the crack length of the healed and virgin samples can be ignored as *P*_*c*_ remains constant along the length of the sample^28^. Mechanical testing of the virgin and healed materials was carried out to obtain values for *P*_*c*_ and the values inserted into the following equation:

2 $$Self-healing\,efficiency=(\frac{\alpha {P}_{{c}_{healed}}}{\alpha {P}_{{c}_{virgin}}})\times 100 \% $$

Assuming that the geometric constant *α* remains the same for the virgin and healed material, it can be cancelled from equation (2) to give:

3 $$Self-healing\,efficiency=(\frac{{P}_{{c}_{healed}}}{{P}_{{c}_{virgin}}})\times 100 \% $$

**Figure 3:**
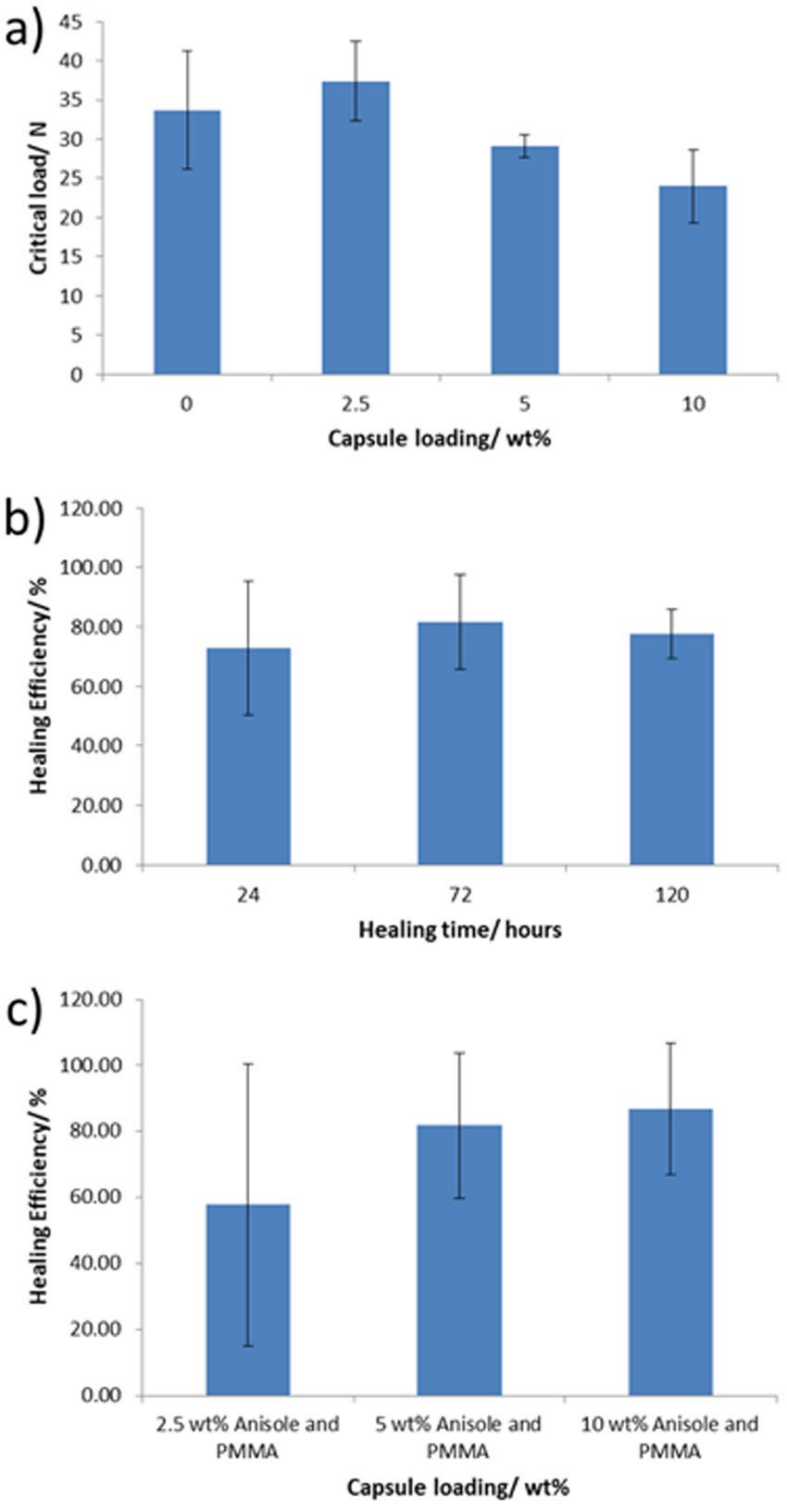
Bar charts showing (**a**) the effect of capsule loading on the critical loadings, $\frac{{K}_{c}}{\alpha }$. Tests were performed in triplicate – error bars denote the standard deviation; (**b**) healing efficiencies of 5 wt% capsule loaded samples with different healing times. Samples were healed at 25 °C for 24, 72 and 120 hours; (**c**) healing efficiencies of samples with different capsule concentrations. Samples were healed at 25 °C for 72 hours. The tests all were performed in triplicate – error bars denote the standard deviation.

The molded TDCB samples were pre-cracked and loaded into a universal testing instrument to perform mode I tensile fracture testing (Figs S9). Although the exact value of *α* is unknown for this material, we can study the relative effect of capsule loading on composite *K*_*c*_ at different microcapsule loadings by plotting the experimentally obtained values of *P*_*c*_, which is equivalent to $\frac{{K}_{c}}{\alpha }$, against capsule loading. We observe that the presence of capsules within the matrix increases the composite fracture toughness, although the effect plateaus and no increase in fracture toughness was observed by increasing the capsule loadings beyond 2.5 wt% (Fig. 3a). The presence of tail-like structures in the wake of the microcapsules in the fracture plane (Fig. S3) suggests that crack pinning contributes to the fracture toughening^29^. Hackle markings, which tend to form during violent fracture when both plastic deformation and branching of the crack front occur^30^, can also be observed. Both the tail and hackle markings increase the surface area of the crack plane, and thus the energy absorbed by the composite during crack growth, thereby increasing the resulting fracture toughness. This fracture toughening mechanism as a result of incorporation of urea-formaldehyde capsules is supported in the literature for a number of materials including epoxy resins^29^ and thermoplastics such as PMMA^22^.

As outlined in Equation 2, the healing efficiency could be calculated through mechanical testing of the TDCB specimens. After the initial fracture event, samples were allowed to heal in a temperature regulated environment at 25 °C for 24, 72 and 120 hours to determine the optimum healing time. The healing efficiencies of samples with 5 wt% capsule loading were investigated (Fig. 3b) and on average, the healing efficiency appears to reach a maximum after 72 hours.Keeping the healing time at 72 hours, the capsule loading was then increased in order to ascertain whether increasing the capsule concentration could achieve higher healing efficiencies. Capsule loadings of 2.5, 5 and 10 wt% were investigated; all samples were healed for 72 hours at 25 °C (Fig. 3c). In all samples some healing was observed, with higher healing efficiencies achieved at higher loading rates. In this work, a maximum healing efficiency of 87% was achieved at a capsule concentration of 10 wt%. However, at 10 wt% capsule loadings, we observe a drop in the fracture toughness of the composite (Fig. 3a) from that at 5 wt% capsule loading. Therefore, the healing efficiencies for composites with higher loadings of microcapsules were not investigated.

**Figure 4:**
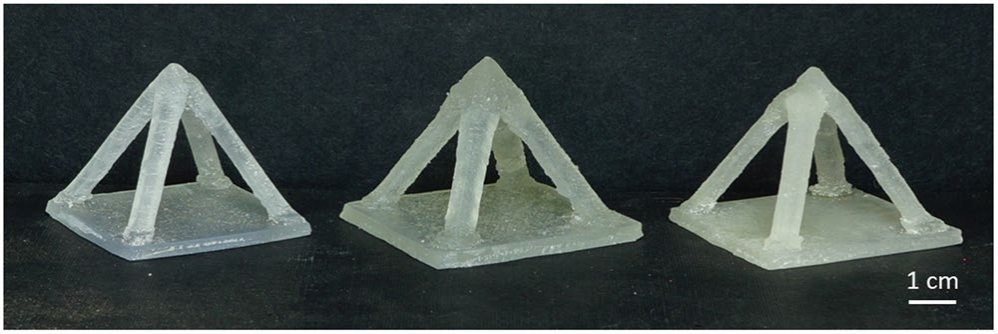
Photograph of 3D printed objects with various capsule loading values. From left to right the microcapsule loading increases from 0 to 5 and then 10 wt%.

**Figure 5:**
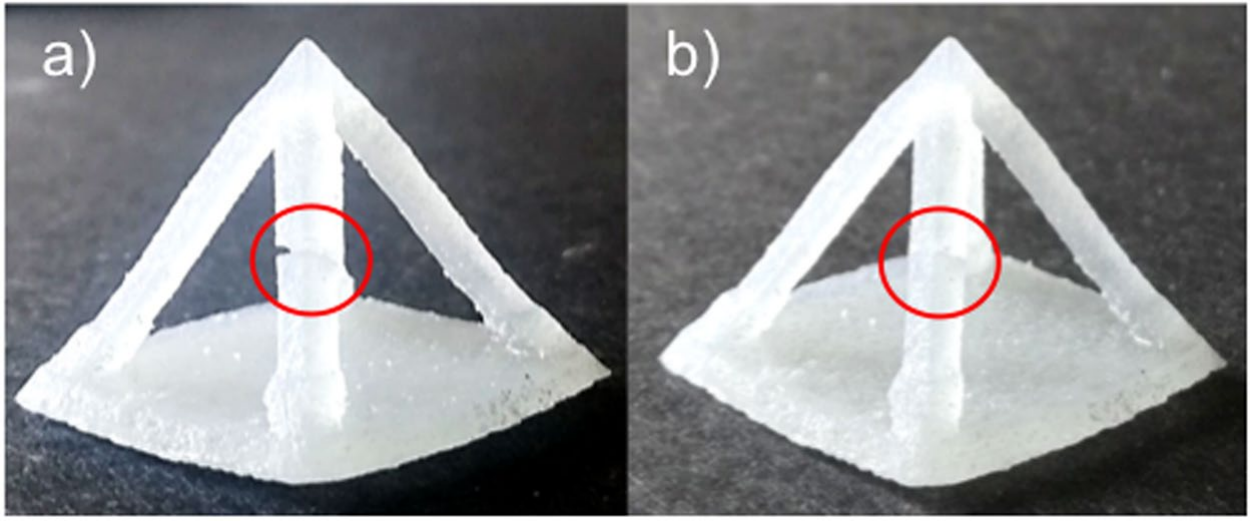
Photograph of (**a**) A 3D printed sample which contains 5 wt% anisole with PMMA capsules. The cut is highlighted in the red circle; (**b**) the 3D printed sample after the two fracture planes were pushed back together and allowed to heal for 3 days at 25 °C. The healed section is highlighted by the red circle.

To demonstrate 3DP of extrinsically self-healing composites, resins with 0, 5 and 10 wt% loadings of microcapsules were poured into printer resin trays and printed by an SL 3D printer (Form 1+ , Formlabs, USA). Addition of the microcapsules to the resins caused the originally clear resin to appear cloudy. However, we observed no noticeable effect on print quality due to light scattering by the capsules (Fig. 4). This observation is supported by the fact that many commercial SL 3DP resins contain light-scattering particulates and pigments, giving them high turbidity, whilst still affording high quality prints. To test the healing of 3D printed samples, damage was inflicted onto the samples and the damage monitored. We found that the release of microcapsule contents onto the fracture planes allowed the healing of cracks and breakages (Fig. 5). Therefore, by incorporating anisole/PMMA containing microcapsules into the resins, objects with self-healing ability can be 3D printed with these resins.

The ability to directly 3D print structures with extrinsic self-healing characteristics has, to our knowledge, not been reported prior to this work. Previous reports on 3D printed self-healing materials are based on intrinsically self-healing materials and their direct-ink writing. However, direct-ink writing based 3DP requires significant optimization of ink rheology, which will differ for each formulation, thereby presenting significant complications^13,14^.

In summary, we have demonstrated SL 3DP of a solvent-welding based self-healing material through addition of self-healing capsules to commercially available resins. The healing efficiency of the work showed a maximum recovery of 87% with respect to the critical load, tested by mode 1 fracture toughness. Further investigation could improve the self-healing efficiency of this material through exploration of different solvents and encapsulated polymers to enhance the solvent-welding mechanism. These results are promising for applications requiring materials with bespoke structures as well as extended structural integrity, such as in personalized medicine. For example, researchers have started to explore the use of solvent and PMMA containing urea-formaldehyde microcapsules to improve the lifetime expectancy of bone cement^23^. The ability to combine this with 3DP would further improve the prospect of such materials being utilized within this field, particularly with the rapid development of commercial biocompatible resins. Further, our approach of adding microcapsules to rapidly incorporate functionality to readily available commercial inks is attractive due to its ease of adoption and flexibility. This promising approach has widespread applications that can be easily modified to incorporate alternative functionalities to 3D printed materials, such as for hollow glass sphere containing light-weight composites^31^, or for flame retardant composite materials; we will therefore investigate such alternative applications in future work.

## Methods

### Materials

The Photocentric UV Laser Hard Clear resin (PUHC resin) was provided by Photocentric 3D. Poly(methyl methacrylate) (MW 120,000), 37 wt% formaldehyde solution in water, ammonium chloride 99.5%, poly(ethylene-alt-maleic anhydride) (Mw 100 kDa – 500 kDa) were purchased from Sigma Aldrich. Anisole (99%), urea (98%) and resorcinol (99%) were purchased from Alfa Aesar. Acetone and sodium hydroxide pellets were purchased from VWR and Fisher Scientific, respectively. All reagents and solvents were used as received. For a detailed description of methods used, please refer to the ESI.

### Capsule synthesis

Urea (2.5 g), resorcinol (0.25 g) and ammonium chloride (0.25 g) were dissolved in a 0.5 wt% ethylene-maleic anhydride copolymer (EMA) solution (125 mL). The pH was adjusted to 3.5 by addition of saturated sodium hydroxide solution. The solution was mechanically stirred with a 3-pitched blade propeller (d = 50 mm, bore = 8 mm, purchased from Cole – Palmer) at 400 rpm. 5 wt% PMMA in anisole (30 mL) was added to the solution during stirring, with the resulting emulsion allowed to stabilise for 10 min. Formaldehyde solution (6.39 g) was added and the solution covered and heated for 4 h at 55 °C with the same stirring parameters. The resultant capsules were washed with acetone (3 × 5 mL) and filtered under reduced pressure, then air-dried for 24 h to yield the free-flowing urea-formaldehyde self-healing capsules (UF-SHC). The capsules were size selected using 300 μm and 38 μm laboratory sieves to isolate the target size range.

### Preparation of 3D prints and mechanical testing samples

2.5, 5 and 10 wt% formulations of UF-SHC in the PHC resin were prepared by adding the required capsules to the resin and mixing by hand with a spatula for 2 min to distribute the capsules. The mixture was then degassed via vacuum to remove any air that was incorporated into the mixture during mixing. The degassed mixture was then poured into the resin tank of a Formlabs 1 + SLA printer. An STL. file of a desired structure was then loaded into the Preform software, produced by Formlabs, in order to generate a G.code. A layer height of 200 μm was selected. The generated G.code was then read by the printer to produce the desired print via layer-by-layer photocuring. The finished print was removed from the build plate and washed with isopropanol and water to remove any un-polymerised resin on the outer surface.

For the purposes of mechanical testing, the prepared formulations were poured into silicone molds and cured under UV light at 365 nm.

## Electronic supplementary material

Supplementary Information
