# Supplementary material for: Stereolithographic 3D printing of extrinsically self-healing composites
Source: Sci Rep. 2019 Jan 23;9:388. doi: 10.1038/s41598-018-36828-9 (PMC6344598; doi:10.1038/s41598-018-36828-9)
Supplement: Supplementary file 1 — Supplementary Information [file 41598_2018_36828_MOESM1_ESM.pdf]

Supporting Information

Stereolithographic 3D Printing of Extrinsically Self-healing Composites

Polly Sanders,[a] Adam J. Young,[a] Yang Qin, [a] Kevin S. Fancey,[a] Michael R. Reithofer,[b] Rémy Guillet-Nicolas,[c] Freddy Kleitz,[c] Nicole Pamme, [a] Jia Min Chin* [a b]*

a. University of Hull, Cottingham Road, HU6 7RX, United Kingdom.

b. Institute of Inorganic Chemistry, Faculty of Chemistry, University of Vienna, Währinger Straße 42, 1090 Vienna, Austria.

c. Department of Inorganic Chemistry – Functional Materials, Faculty of Chemistry, University of Vienna, Währinger Straße 42, 1090 Vienna, Austria

Email address: J.Chin@hull.ac.uk

Email address: Michael.reithofer@univie.ac.at

Contents

Methods **2**

Optical microscopy and capsule size analysis **2**

Scanning electron microscopy, SEM **4**

Fourier transform infrared spectroscopy – attenuated total reflectance, FTIR-ATR **5**

Thermogravimetric analysis, TGA **7**

Self-healing analysis **9**

Anisole Penetration Photographs **11**

References ***12***

Methods

Optical microscope images of capsules were captured using an Olympus BX50 optical microscope fitted with an Infinity X camera and x4 magnification for size analysis. Scanning electron microscope (SEM) pictures were captured by a Zeiss Supra 55 VP electron microscope. Samples were sputtered with gold prior to imaging. FTIR-ATR was carried out on a Thermoscientific Nicolet iS5 fitted with a Pike Miracle diamond ATR attachment. Thermogravimetric analysis was carried out on a Mettler Toledo TGA/SDTA851^{e} equipped with a TS0801RO autosampler. Samples were heated at a rate of 10 °C·min^{-1} from 25 to 900 °C under a 50.0 mL·min^{-1} nitrogen flow. Mechanical testing was carried out using an EZ 50 universal testing machine from Lloyd instruments using a 100 N load cell.

Optical microscopy and capsule size analysis

From the optical microscope image below (Figure S5), the roughness of the capsules can also be observed. However, capsule roughness is less discernible using optical microscopy compared to SEM (Figure 2a). This is due to the low contrast, and the narrow depth of focus relative to capsule height. The images were recorded on a BX 50 Olympus microscope using either a x4 or a x20 magnification objective. This setup results in a depth of focus of either 175 or 6 µm respectively, which can be calculated from the Berek formula.[1] With the x4 magnification, capsule roughness may not be very obvious due to the low magnification. With the x20 magnification, the low depth of focus (6 µm) relative to the capsule height of more than 200 µm means that most of the capsule is out of focus during optical microscopy. SEM was therefore performed to complement optical microscopy.

The average capsule diameter was investigated through analysis of optical images (Figure S6). The capsules were polydisperse, however some degree of control can be gained by adjusting the stirring speed of the propeller during synthesis (Figure S7). The optimum stirring speed was determined to be 400 rpm to yield capsules with an average diameter of 130 ± 15 µm. For samples prepared at each stirring speed, 100 capsules were measured using the ImageJ analysis software.

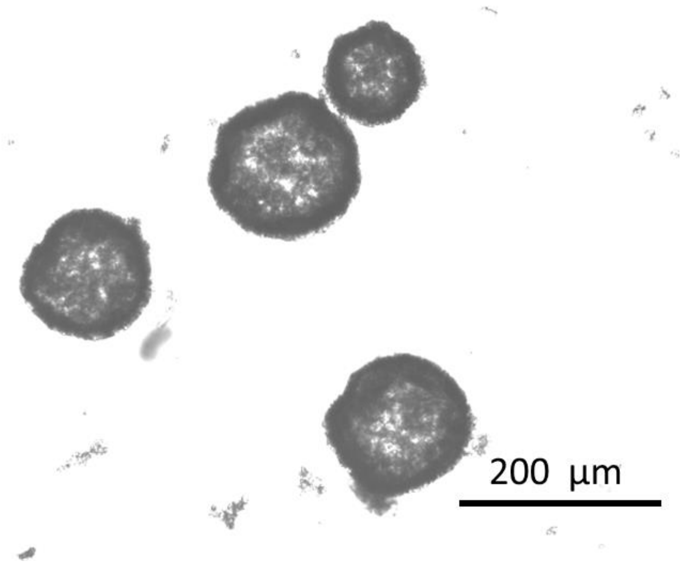

Figure S1: Optical microscope image of the microcapsules produced using a stirring speed of 400 rpm. Capsule sizes were measured to be 130 ± 15 µm in diameter (n= 100).

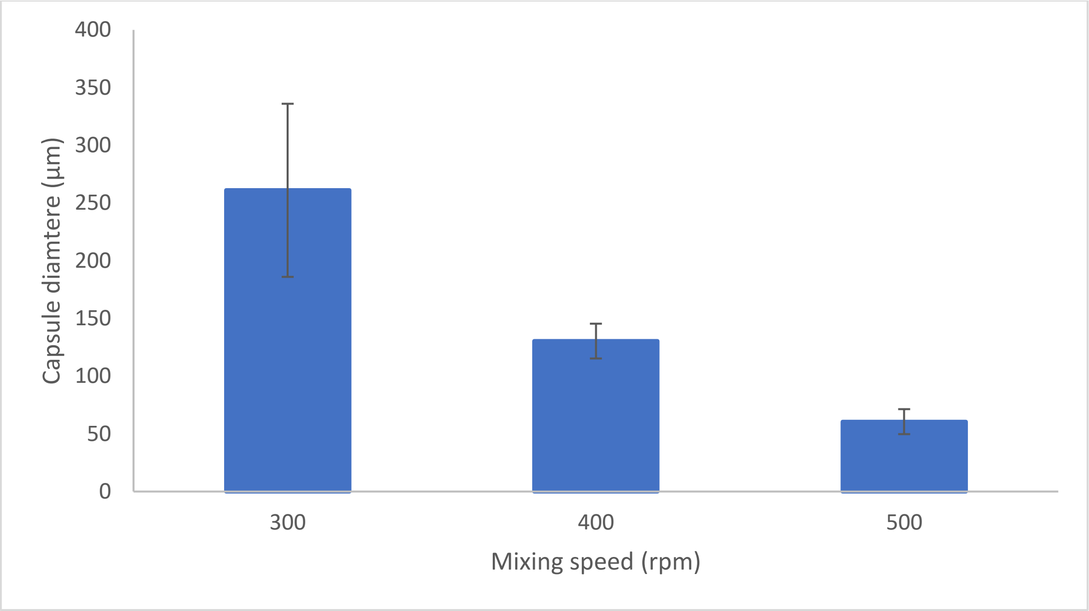

Figure S2: Size distribution of capsules generated using various mixing speeds. Error bars relate to 1 standard deviation (n=100).

Scanning electron microscopy, SEM

The morphology of the capsules studied by SEM showed spherical capsules with a rough surface. The shell cross-section was investigated by crushing the capsules using a pestle and mortar and washing thoroughly with acetone to yield fragments of the empty shells (Figure 2b).

Samples with the capsules embedded within the polymer were also prepared. The cured polymer with embedded capsules was fractured and the fracture plane washed with isopropanol to remove any anisole that escaped the ruptured capsules. Upon observation of the fracture plane, voids can be seen where the capsules were present within the polymer. Striations are present in the fracture plane creating a tail-like appearance to the voids (Figure S3). These tail markings suggest that crack pinning may act as a toughening mechanism in the composite material.

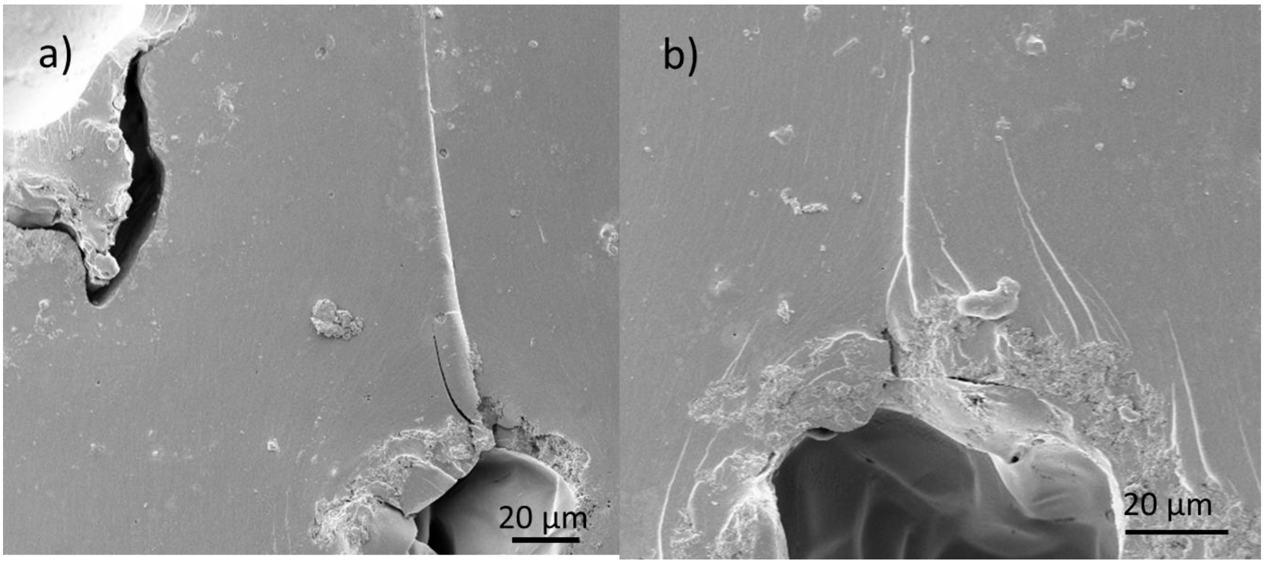

Figure S3: SEM image showing the a) tail marks in the wake of the microcapsules, which are suggestive of a toughening effect upon capsule loading and b) hackle markings around the microcapsule

Fourier transform infrared spectroscopy – attenuated total reflectance, FTIR-ATR

Samples were directly loaded onto the diamond window of the ATR instrument for characterisation. Figure S4 shows the cured resin, which is a mixture of acrylates, with the peaks identified to support this. Figure S5 shows the anisole/PMMA filled urea-formaldehyde capsules. Figure S6 shows the spectrum of 5 wt% capsule loadings in the cured resin. Increasing the capsule loading saw no distinctive changes in the spectra, as the IR bands of the resin matrix swamp the spectra of the composite.

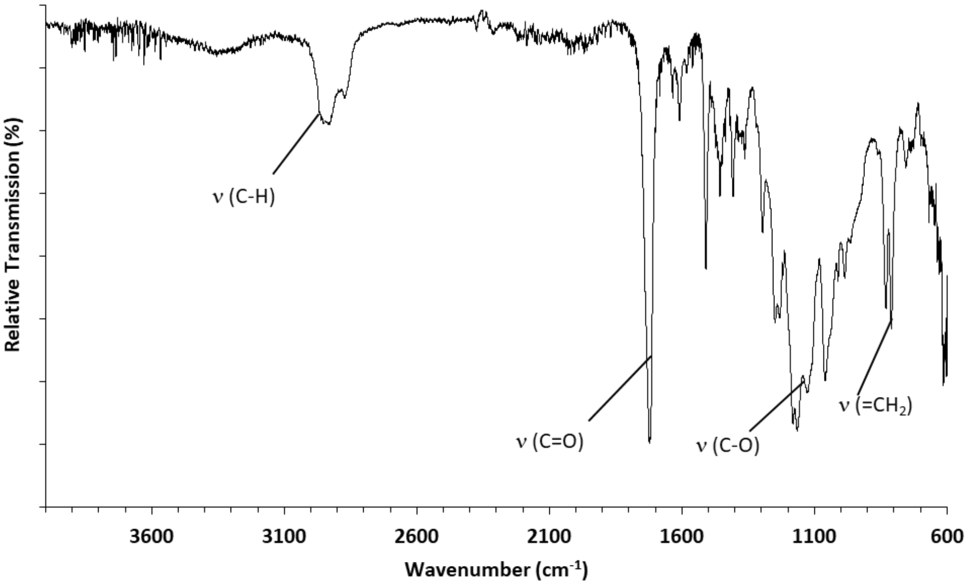

Figure S4: Diamond ATR spectra for the cured commercial Photocentric UV Hard Clear (PUHC) resin.

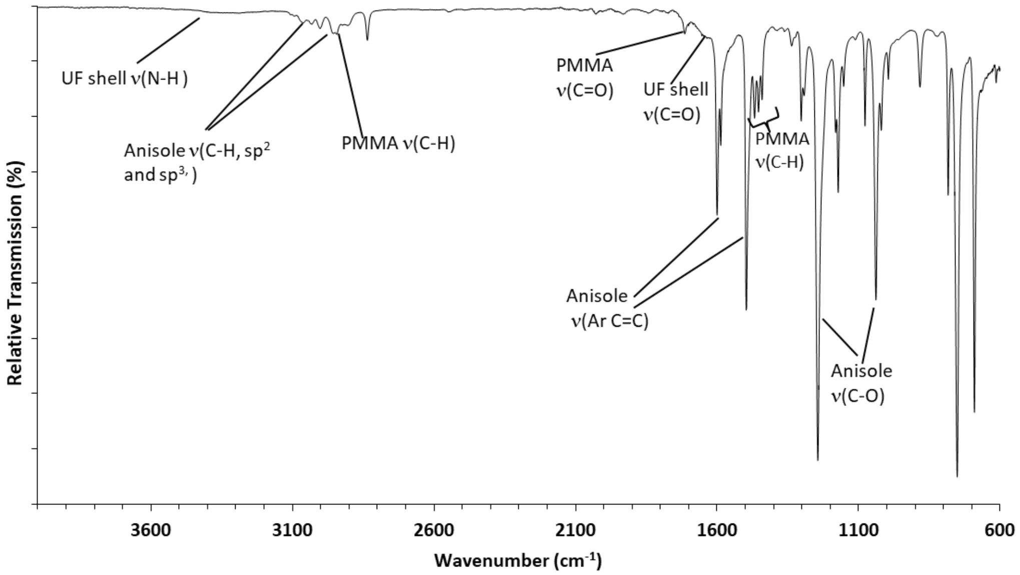

Figure S5: Diamond ATR spectra of PMMA/anisole urea-formaldehyde (UF) microcapsules.

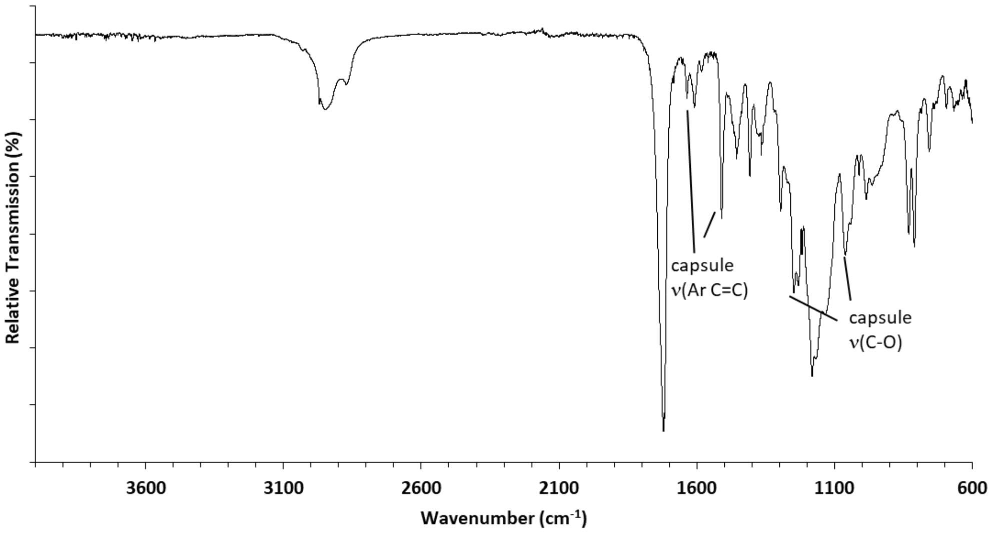

Figure S6: Diamond ATR spectra of 5 wt% capsules in cured PUHC resin, where some of the strong IR bands from the microcapsules can be discerned

Thermogravimetric analysis, TGA

TGA was performed on capsules containing anisole and 5 wt% PMMA core, cured PUHC resin as well as cured PUHC resin with 5 wt% microcapsule loadings separately.

During analysis of the capsules as shown in Figure S7, the weight loss at 200-240 °C indicates the decomposition of the urea formaldehyde shell.[2,3] This, together with vaporization and expansion of capsule contents (boiling point of anisole is 154 °C), caused the capsules to explode during each of several TGA attempts. The violent reaction led to movement of the TGA pan and inaccurate data. To mitigate this, a slower heating rate of 5 °C·min^{-1} and a very small amount of sample (0.9 mg) was utilised for TGA of the capsules, which reduced the impact of the solvent expansion. The noise present is attributed to thermal drift and to the smaller sample size being used. Nevertheless, this afforded an estimation of percentage weight remaining at 420 °C of 11.9%.

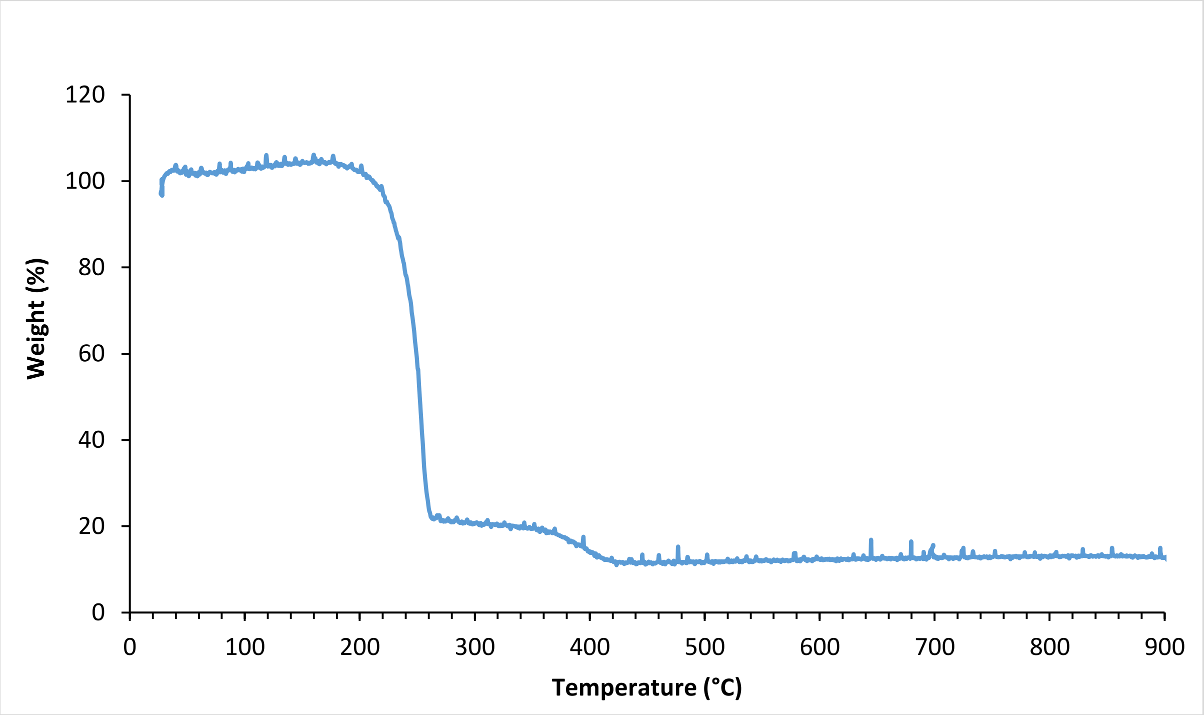

Figure S7: TGA of PMMA/anisole-containing urea-formaldehyde microcapsules.

TGA analysis of the resin with and without capsules is shown in Figure S8. For the resin without capsules, the percentage weight remaining at 420 °C is 75.2%. For the resin with 5 wt% microcapsule loading, at 400 °C, the percentage weight remaining is 71.3%, which is a close fit to the expected value of 71.5% (given by 75.2% x 0.95 + 11.9% x 0.05). Both the pure resin and the 5 wt% capsules samples showed the largest drop in weight loss at 420 °C and 500 °C which has been attributed to the degradation of the matrix resin.

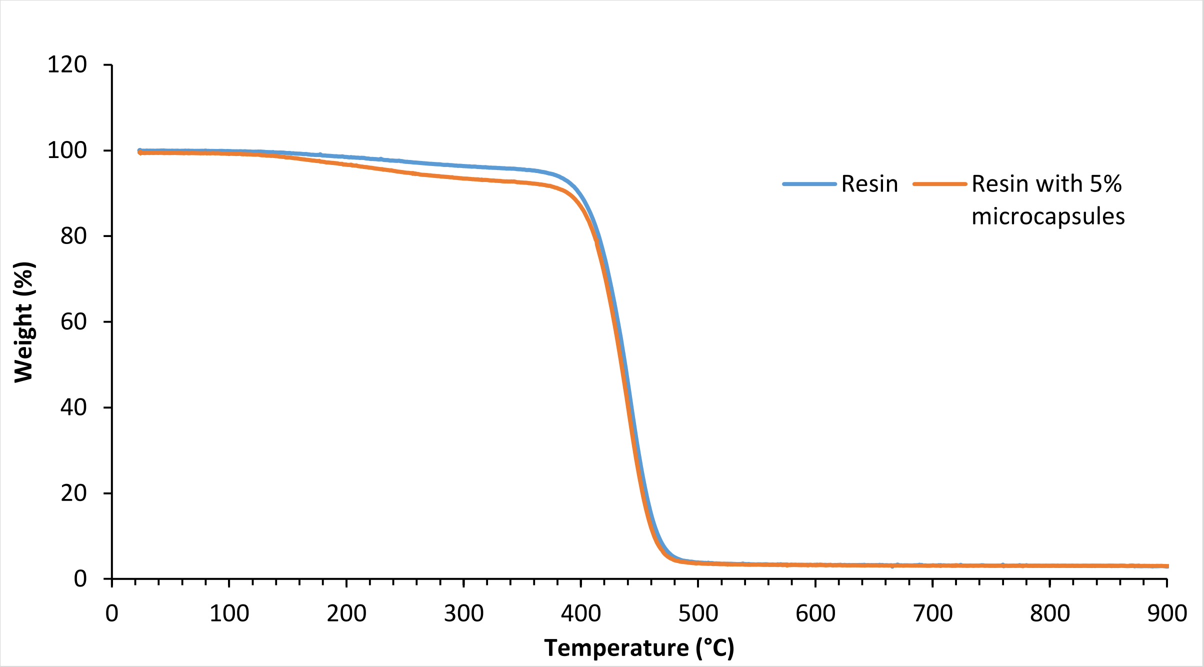

Figure S8: TGA of the 0 and 5 wt% formulations, the urea-formaldehyde solvent loaded microcapsules (red) and the cured 3DP resin (grey).

Self-healing analysis

Mechanical testing of the virgin and healed materials was achieved by pre-cracking samples within the notched section of the samples, loading into a universal testing instrument and performing mode 1 tensile fracture testing using a tapered double cantilever beam (TDCB) geometry, illustrated in Figure S9.

The required geometry was first generated as a 3D model and then printed in poly(lactic acid) (PLA) using an Ultimaker 2+ 3D printer to generate a master, from which the 2-part silicone moulds were cast. The silicone moulds were prepared using the Sylgard 184 kit that was mixed according to the manufacturer's instructions and degassed via centrifugation at 10,000 rpm for 5 min. The formulations were cured in the moulds to achieve the desired geometry through utilisation of a 365 nm wavelength UV light source at a fixed distance of 15 cm from the curing stage.

The displacement rate was fixed at 0.5 mm min^{-1} for all tests and the propagated crack lengths ranged from 15-20 mm. After the initial fracture event, the self-healing samples were unloaded, and the crack surfaces allowed to come back together. The samples were then left to heal at 25 °C for 24 to 72 h before being reloaded and undergoing fracture again. An example of the load displacement curves that were produced is shown in Figure S10. From this the % healing efficiency was calculated from the ratio of the critical loads of the virgin and healed samples. The tests for each condition was performed in triplicate, with sets of 3 samples.

For the 3D printed samples, the leg of a 5 wt% capsule loaded sample was cut (Figure 5a). The two fracture planes were then pushed together in order to ensure that close contact of the two surfaces was maintained during healing. After 3 days at 25 °C, the two surfaces had re-joined to heal the fracture (Figure 5b).

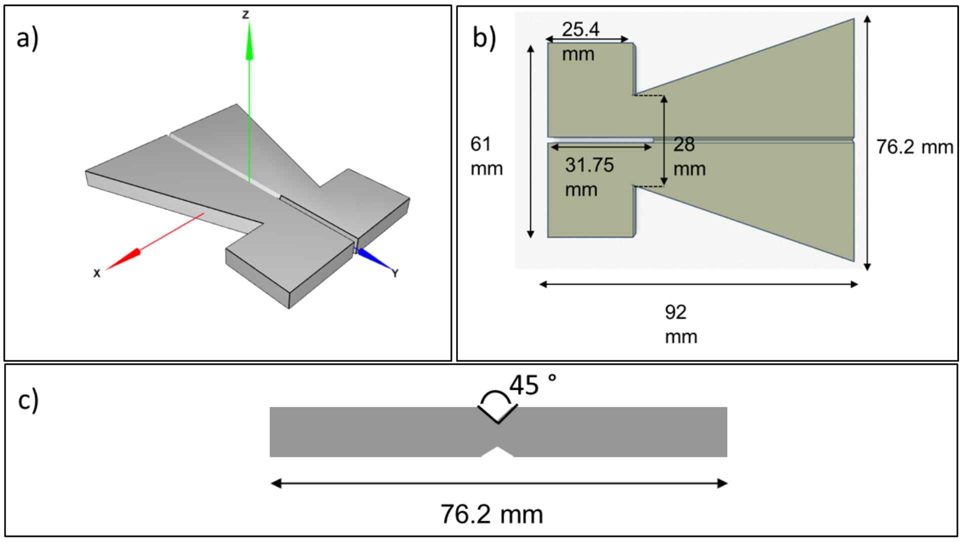

Figure S9: Sample geometry for TDCB specimens. a) 3D graphical representation of the TDCB sample. b) The view of the samples from above with the dimensions of the samples. c) The view of the sample from the side which shows the 45° angle grooves on the top and bottom of the samples.

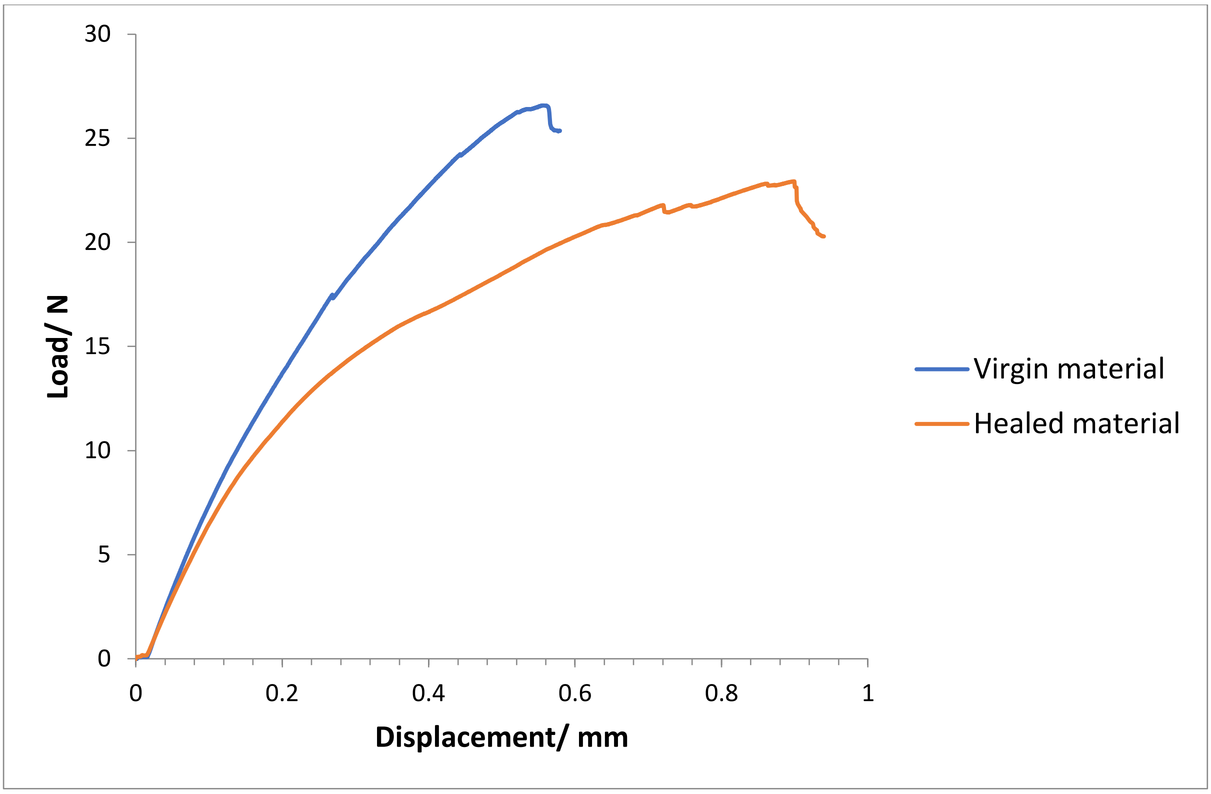

Figure S10: An example of the load displacement curves acquired from the virgin and healed samples (from the fracture plane).

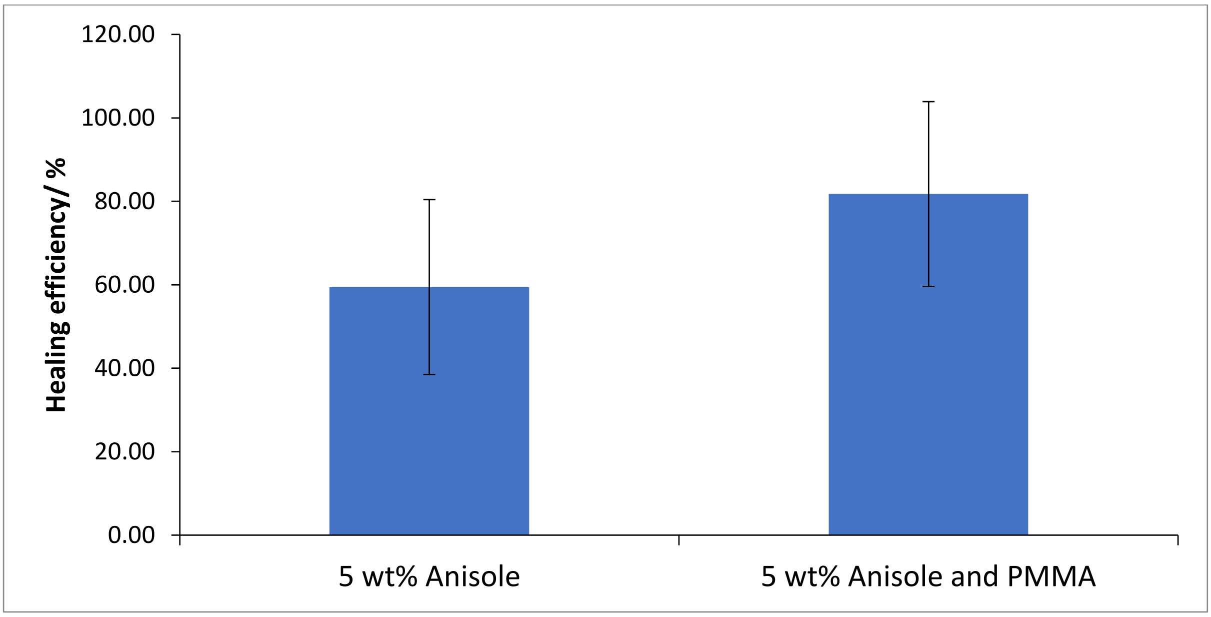

Figure S11: Healing efficiency graph of cured resin containing capsules with and without PMMA. Experiments were performed in triplicate.

Anisole Penetration

Samples of cured resin have been exposed to anisole in order to show that anisole can penetrate into the resin. A drop of anisole was added to the surface of the cured resin and left to penetrate the resin sample. A dye, tetraphenyl porphyrin, was added to the anisole to provide a visual representation of the path of the anisole through the polymer.

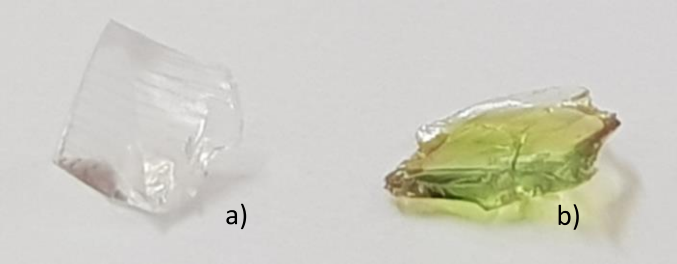

Figure S12: Photograph cured photocentric hard clear resin a) without anisole and b) with anisole (dye added to aid visualization)

References

1 Olympus. *Depth of focus*, <https://www.olympus-ims.com/en/microscope/terms/focal_depth/> (

2 Liao, L. *et al.* Preparation and characterization of microcapsule containing epoxy resin and its self-healing performance of anticorrosion covering material. *Chin. Sci. Bull.* **56**, 439-443, (2011).

3 Shahabudin, N., Yahya, R. & Gan, S. N. Microcapsules of Poly(urea-formaldehyde) (PUF) Containing alkyd from Palm Oil. *Mater. Today: Proceedings* **3**, S88-S95, (2016).
